# Figure skating: Increasing numbers of revolutions in jumps at the European and World Championships

**DOI:** 10.1371/journal.pone.0265343

**Published:** 2022-11-30

**Authors:** Thomas Rauer, Hans-Christoph Pape, Matthias Knobe, Tim Pohlemann, Bergita Ganse

**Affiliations:** 1 Department of Trauma Surgery, University Hospital Zurich, Zurich, Switzerland; 2 Department of Orthopedic and Trauma Surgery, Lucerne Cantonal Hospital, Lucerne, Switzerland; 3 Department of Trauma, Hand and Reconstructive Surgery, Saarland University Hospital, Homburg, Germany; 4 Werner Siemens Foundation Endowed Chair of Innovative Implant Development, Saarland University, Homburg, Germany; University of Belgrade: Univerzitet u Beogradu, SERBIA

## Abstract

Figure skating is associated with a high prevalence of sport-specific injuries and overuse symptoms. Impacts are of greater magnitude in jumps with more revolutions that are thus connected to a greater risk of injury. While figure skating programs seem to have recently increased in difficulty, performance trends have not yet been reported in the literature. We hypothesized increasing performance and decreasing age trends of the best athletes who competed at international level in recent years. Furthermore, we aimed to identify and analyse objective performance parameters and to assess a potential link between age and the risk of injury. The development of the number of double, triple and quadruple jumps in the singles figure skating events at the World and European Championships since the introduction of the new ISU Judging System in the 2004/2005 season was analysed using regression statistics and Student’s T-Tests. In all groups, the numbers of jumps with fewer rotations significantly decreased in the favour of jumps with more rotations. Women only started to perform jumps with four rotations in 2019. In the men, the number of quadruple jumps increased from an average of less than one to more than three in recent years (European and World Championships, both p < 0.001). In the European, but not in the World Championships, the average age increased in the men and decreased in the women (European Championships, men and women p = 0.006). Our study was the first to assess performance trends in elite figure skating. The incidence of injuries and overuse syndromes in figure skating needs to be monitored cautiously, as increases can be expected following recent gains in performance and jump complexity.

## 1. Introduction

Figure skating is a popular winter sport and part of the Olympic Winter Games [1]. It is associated with a high prevalence of sport-specific injuries, especially stress fractures and muscle strains, as well as of overuse symptoms, such as tendinitis, patellofemoral syndrome, and low-back pain [2–5]. In the singles skating events, overuse symptoms are more prevalent than acute injuries, while in pair skating, ice dancing and synchronized skating it is the other way around [6]. The most frequently injured locations are the ankle, knee, tibia and hip/groin [7, 8]. Acute and upper extremity injuries are more frequent in partner disciplines due to the throws and lifts performed [6]. The overall career prevalence of stress fractures was found to be 24% in all athletes, and 33% in those who train 12x or more per week, independent of sex [8]. In another study, the 1-year prevalence of a severe sports injury episode was 31% [7]. Older age and an increased number of skipped meals per week were associated with a sports injury episode [7]. In figure skaters, due to massive impact forces, bone mineral density, trabecular bone mineral density, and bone strength are increased in the landing leg compared to the take-off leg [9, 10].

In multi-revolution jumps, the time for the force to dissipate is shorter when there are more revolutions, while jump height and vertical velocity at take-off do not significantly differ among single, double, and triple revolution jumps [10, 11]. Thus, the impacts are of greater magnitude and more intense in jumps with more revolutions, and associated with a greater risk of injury [10]. Athletes with higher skills generate greater vertical velocities during take-off [11]. Differences between triple and quadruple jumps include the timing of hip and shoulder rotation before toe-pick, increases in rotational velocity with tighter rotating positions for longer durations, and greater vertical velocity due to more powerful extension of the legs [12]. In recent years, programs have increased in difficulty [13]. In line with this, at international elite championships, numbers of revolutions in jumps seem to have increased as well. Due to the increasing difficulty of jumps with more rotations, the number of jumps might serve as an objective parameter of performance and help to study performance trends.

Some authors have speculated about a plateau of human sports performance in elite sports [14–17]. Others reported a slower asymptotic increase in elite peak performance [18]. Declining performance trends were found in elite track and field [19], that might be due to increasingly strict anti-doping regulations [20, 21] or due to changing popularity of sports, i.e. towards extreme sports, climbing, BMX and e-sports [22–24]. Performance trends have not been previously reported for figure skating, but are of interest to evaluate risk trends for injury and chronic strain. Since figure skating is not associated with results in times or distances, as compared to sports such as swimming, cycling or track and field [25–28], other measures need to be found to estimate the athletes’ performance for such an analysis. Therefore, in this study, to study performance in an objective way, the number of turns of the jumps were used as judged by a technical panel based on a restrictive set of technical rules. Falls, under-rotated or downgraded jumps were not considered. Only the definitive jump performance recognized by the technical jury was taken into account and evaluated. In addition to performance or difficulty trends, age trends are of interest to assess risk profiles, especially with regard to the fact that older age was associated with more sports injuries [7]. We hypothesized increasing performance and decreasing age trends of the best athletes who competed at the European and World Championships in recent years.

## 2. Materials and methods

Ethical approval was obtained from the IRB of Saarland Medical Board (Ärztekammer des Saarlandes, application number 135/21). The need for consent was waived by the ethics committee.

### 2.1 Regulations

The World and European Championships are regulated by the International Skating Union (ISU) [29]. In the season 2004/2005, the new ISU Judging System replaced the 6.0 scoring system, which had been in place since the 1891 European Championships in Hamburg. We only analysed results since 2005 to guarantee comparability, since the new regulations made jumps with more revolutions more interesting for athletes. During competition, scoring is conducted by combining a base value with the grade of execution (GOE), which results in the combined technical elements score (TES) [30]. As the GOE is a subjective score that might differ among judges, we chose to analyse the number of rotations in the jumps performed as an objective measure. The number of turns as well as the correct execution of the jumps are assessed during the championships by the technical panel. The technical panel consists of two technical specialists and a technical controller. Evaluation is conducted on the basis of a restrictive set of technical rules, so that an objective evaluation of the technical execution and the number of rotations performed is ensured. In figure skating, the official terminology uses the term ‘Ladies’ (L) for women and ‘Men’ (M) for men.

### 2.2 Jumps and turns

The jumps are the outstanding features of the individual disciplines. We chose this approach, because the difficulty of the jumps increases with the number of rotations in the air. Thus, the number of rotations not only reflects the performance level in general, but also the increasing forces acting on the athlete’s body. The following jump types exist in the singles figure skating: Toe Loop, Loop, Salchow, Flip, Lutz and Axel. Each of these jumps can be executed with two, three or four rotations. Seven jumps and no more than two identical ones are to be delivered by each athlete during competition. The number of jumps, jump sequences and jump combinations is determined by the current scoring system of the International Skating Union Special Regulations & Technical Rules. This system provides details on the scoring value of each element that varies with the way elements and jumps are combined. It also specifies how many jumping elements a well-balanced Free Skating program must contain. Whether these jumps are performed as double, triple or quadruple jumps is up to the athletes according to their ability and the choreography of the program. Athletes adapt to the current rules by weighing the benefits of jumping against the risks, taking into account their own preferences and personal skills.

### 2.3 Figure skating disciplines

On international level, the five disciplines in figure skating are the ladies’ singles, men’s singles, pair skating, ice dance, and synchronized skating. The broad spectrum of requirements in figure skating includes jumps with multiple revolutions in the air in the individual performances of the single ladies and men, acrobatic lifting and throwing elements in pair skating, as well as aesthetic, athletic dance performances in ice dancing and in the synchronized group performances of up to 20 skaters in synchronized figure skating. Among the Olympic disciplines, we analysed the singles in the present study, as the other disciplines contain only a limited number of jumps due to differences in the scoring system and performance evaluation. In addition, the kinematic of the throw jumps in pair skating differ from the jumps in the singles.

### 2.4 Statistical analyses

The datasets analyzed for this study are third party data and can be found on the website of the International Skating Union (ISU): https://www.isu.org/figure-skating/entries-results/fsk-results [29]. Via this URL, others can access these data in the same manner as the authors. The authors did not have any special access privileges that others would not have.

We assessed the number of double, triple and quadruple jumps via the results databases of the respective international championships, which are freely accessible via the ISU website [29]. For each year and sex, the age and number of jumps with multiple revolutions of the best five athletes were used for analyses. The different years for the analysis of jumps and age are due to the different availability of data. We chose to analyze the best five athletes of each competition and group only, as the differences in performance are huge between the top athletes and the lower-ranking ones. IBM® SPSS® Statistics version 27 was used for statistical analysis. Normal distributions of data were tested by the Kolmogorov-Smirnov and Shapiro-Wilk tests. All data were normally distributed. Regression statistics were performed to compute the slope and coefficient of determination for changes in age and the numbers of jumps over time. To analyse for time-differences in age and numbers of revolutions, two-tailed Student’s t-tests were applied. Averages of the best five athletes in each category of each event in each year were calculated. Changes in average age were analysed for the European and World Championships of the years 2000–2020. Differences in numbers of multi-revolution jumps were analysed for the European and World Championships of the years 2005–2019. Effect sizes are reported as Cohen’s *d* (very small *d* = 0.01, large *d* = 0.80, huge *d* = 2.00). Significance was assumed at p < 0.05.

## 3. Results

### 3.1 Age trends

**Fig 1:**
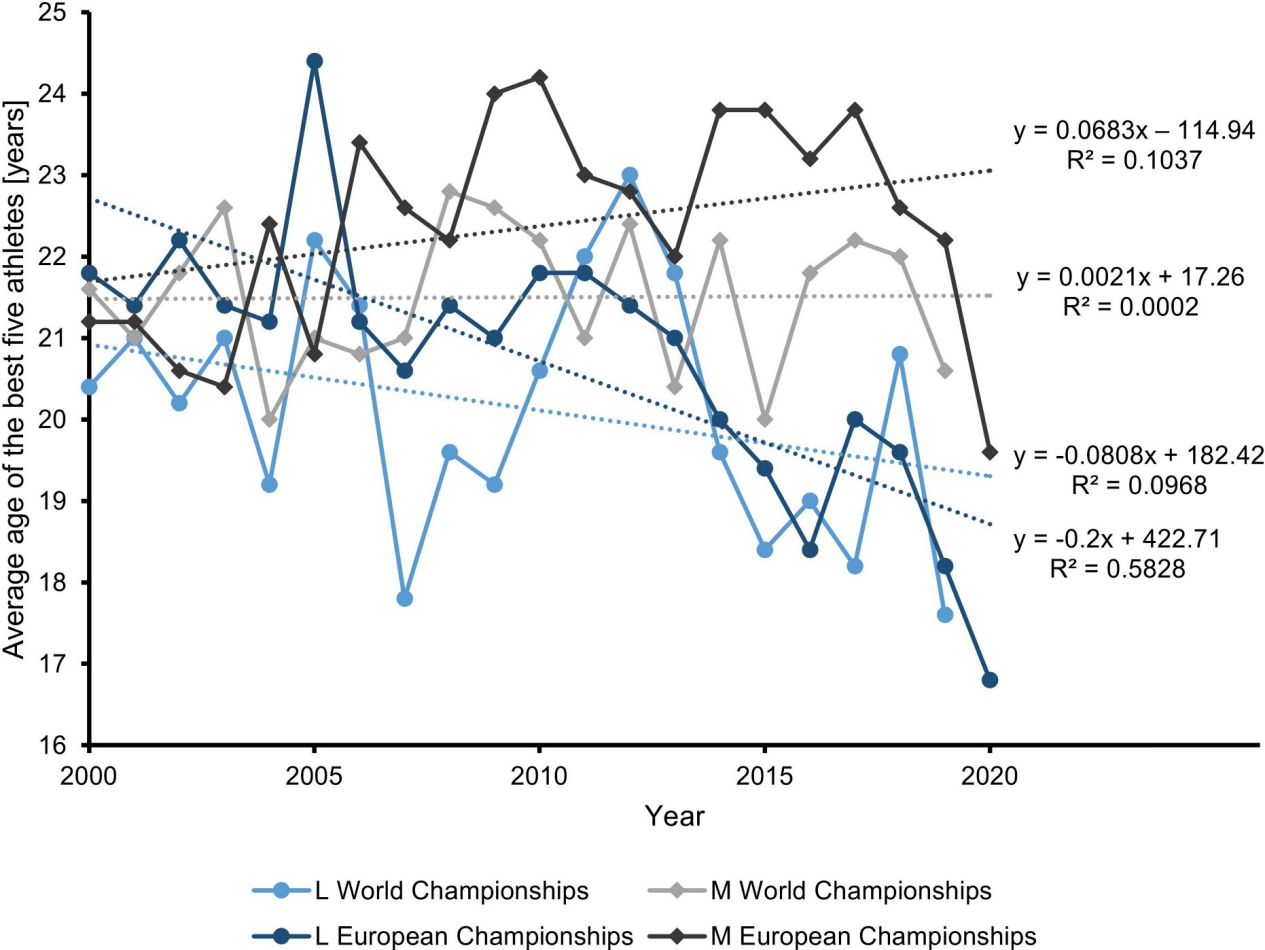
Trends of the average age. Trends of the average age of the best five female (L) and male (M) athletes each who competed in the singles categories at the European and World Figure Skating Championships since the year 2000. Regression analysis was performed to show trends over time. The regression function and the coefficient of determination (R^2^) are shown for each group and indicate high variations in age.

The average age differed significantly between men and women (p < 0.001, *d* = 1.096, men > women, averages: men: 21.9 ± 3.0 years, women: 20.4 ± 3.4 years, data pooled for World and European Championships, years 2000–2020, 21 years). Results of the regression analyses over time and p-values for the statistical tests are shown in **Table 1**. A significant age difference was found between the first and the second decade of the 21st century for the European, but not for the World Championships. This finding was similar for women and men. The average age increased in the men and decreased in the women. **Fig 1** shows the age trends over time and the associated regression parameters spanning both decades. A high age variation was found that is reflected by low R^2^ values as indicated in **Fig 1**.

**Table 1:** Regression slopes of the average age (in years of age per year), the difference (Δ), p-values (one-sided Student’s T-Test), and effect size (*d*).

	Slope 2000–2009	Slope 2010–2019	Δ	p	*d*
L World Championships	-0.172	-0.418	0.246	0.444	0.068
M World Championships	0.080	-0.039	0.119	0.461	0.046
L European Championships	-0.076	-0.440	0.364	**0.006**	1.336
M European Championships	0.301	-0.216	0.517	**0.006**	1.343
**Average:**	**0.033**	**-0.278**	**0.312**		

### 3.2 Numbers of jumps

**Fig 2:**
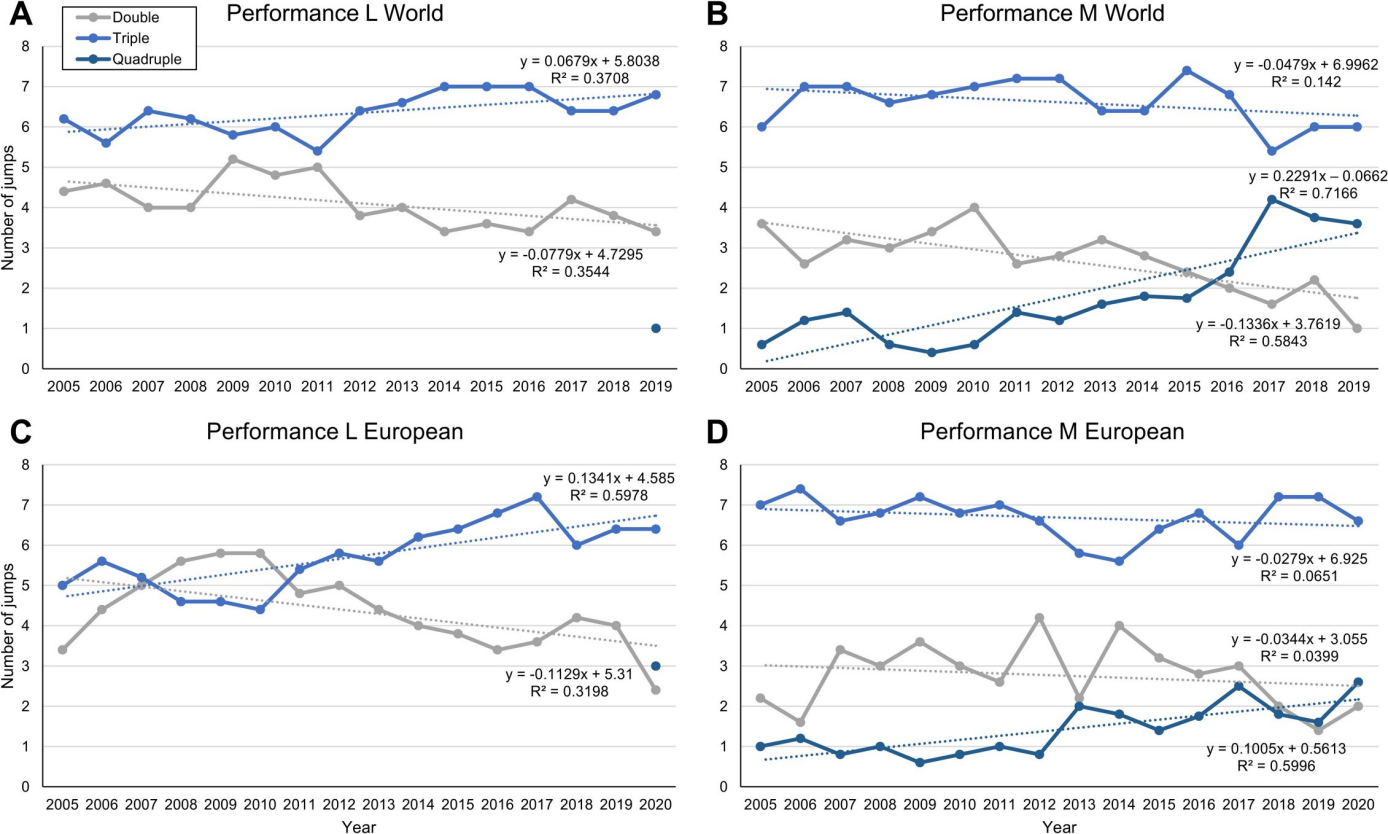
Trends of the average number of double, triple and quadruple jumps. Trends of the average number of double, triple and quadruple jumps of the best five female (L) and male (M) athletes each who competed in the singles categories at the European and World Figure Skating Championships since the year 2005. Regression analysis was performed to show trends over time. The regression function and the coefficient of determination (R^2^) are shown for each group. (A) and (B): World Championships, (C) and (D): European Championships.

In total, 15 World and 16 European Championships were analysed. Women and men performed double and triple jumps, while quadruple jumps in the women only appeared in 2019. The numbers of double jumps decreased significantly over time in both sexes in the World Championships, while in the European Championships, the difference was only significant for the women. In the men, the number of quadruple jumps increased from an average of less than one to more than three in recent years. Results of the regression analyses and p-values for the statistical tests are shown in **Table 2**. **Fig 2** shows the trajectories of the average numbers of jumps and indicates that the numbers of jumps with fewer rotations decreased in the favour of jumps with more rotations.

**Table 2:** Regression slopes (in jumps per year) for each jump type, the difference (Δ), p-values (one-sided Student’s T-Test), and effect size (*d*).

	Jump type	Slope 2005–2011	Slope 2012–2019	Δ	p	*d*
L World Championships	Double	0.121	0.000	0.121	**<0.001**	2.434
	Triple	-0.079	-0.019	0.060	**<0.001**	2.533
M World Championships	Double	-0.033	-0.307	0.274	**0.005**	1.656
	Triple	0.114	-0.143	0.257	**0.042**	0.705
	Quadruple	0.031	0.441	0.410	**<0.001**	2.246
L European Championships	Double	0.279	-0.190	0.469	**0.002**	1.865
	Triple	-0.064	0.087	0.151	**<0.001**	2.793
M European Championships	Double	0.150	-0.257	0.407	0.189	0.486
	Triple	-0.021	0.117	0.138	**0.033**	1.137
	Quadruple	-0.036	0.118	0.154	**<0.001**	3.644
**Average:**	** **	**0.055**	**-0.030**	**0.244**		

## 4. Discussion

The present study analysed numbers of double, triple and quadruple jumps in the singles figure skating events at the World and European Championships to assess trends in performance and age. In the European, but not in the World Championships, the average age increased in the men and decreased in the women. In all groups, the numbers of jumps with fewer rotations decreased in the favour of jumps with more rotations. Women and men both performed double and triple jumps, while women only started to perform jumps with four rotations at international elite championships in 2019. In the men, the number of quadruple jumps increased from an average of less than one to more than three in recent years. Our study was the first to assess and show performance trends in elite figure skating in the ladies’ and men’s singles categories.

### 4.1 Recent increases in performance

The present study detected much more pronounced increases in figure skating performance when compared to data from sprint and distance running [16], while a plateauing of performance was reported in other sports [14–17]. In the past three years, increasing numbers of female ice skaters have started to show quad jumps in competition. Similarly fast increases in performance as in the ladies’ figure skating have been observed in pole vault when it became Olympic for women [31]. The main reason for the exceptional performance increase in figure skating, which is reflected in greater numbers of rotations in jumps, seems to be that recent changes in regulations led to a greater incentive for more complex jumps. In addition, technical improvements in skate development, as well as innovations in training and preparation have likely contributed. Technical improvements include custom-made skates with better stability support for the ankle joints and increasing comfort despite a reduction in weight. Articulated figure skates were invented and tested with the aim to reduce impact forces and the incidence of injury [32]. However, they were not accepted by skaters and eventually discontinued after a few years. In addition, improvements of figure skating rinks and arenas, such as better ice and air quality might have led to more athlete-friendly conditions [33, 34]. With regard to age, increases in the average age of the men suggest that they should be particularly monitored for injuries, since older age was found to be connected to more sports injuries [7].

### 4.2 Implications for injury and overuse symptoms

As jumps with more rotations are associated with impacts of greater magnitude and intensity, the recent developments found in the present study should be monitored concerning injury rates and chronic degenerative damage in athletes. This is especially true for the women who reach their peak performance level rather earlier than later as compared to before. The particularly high incidence of overuse injuries in the single disciplines, among other things, is due to their increasing technical difficulty with jumps that are more complex and connected to longer training times [6]. To achieve the increased level of technical difficulty and to avoid injury, skaters are required to continue to develop their physical skills such as agility, strength and power [11]. In addition, strengthening of the feet and ankles, as well as the promotion of lower extremity flexibility may prevent many of the common lower extremity injuries [6]. As jumps with more rotations are associated with impacts of greater magnitude and intensity, these recent developments should be monitored about injury rates and chronic degenerative damage in athletes, especially in the women who reach their peak performance earlier than before.

### 4.3 Implications for the improvement of training

Training exercises that emphasize eccentric and centric muscle actions and that can be adapted to asymmetric or unilateral movements are a critical component of off-skating training programmes for figure skaters [11]. Progress has been made in the development of off-ice strength and conditioning training, with an increasing emphasis on the importance of core and upper body strength exercises [11]. For stable performance development toward more triple and quadruple jumps, it is essential that off-ice training includes exercises specifically designed to strengthen the abdominal and core muscles so that young athletes can compensate for the forces encountered on the ice during jumps and landings, thus preventing acute injuries and overuse symptoms. Altitude training, by a reduction of air resistance, is known to affect performance in sports involving high velocities and technical skill components, such as ski jumping, speed skating, figure skating and ice hockey [35]. In addition, nutrition is of great relevance for winter sport athletes and plays a crucial role in figure skating, where attention should be given to an increased energy expenditure, accelerated muscle and liver glycogen utilization, exacerbated fluid loss, and increased iron turnover [36].

### 4.4 Methods to analyse performance in figure skating

In figure skating, a median rank aggregation system is used to determine medal winners that is susceptible to idiosyncratic ratings by individual judges [37]. To analyse the athletes’ performance trends in the most objective way, we chose only to use measures that are independent of judges’ ratings. The number of revolutions in a jump is a suitable parameter that reflects performance, as jumps with more rotations are more difficult than jumps with less rotations, since higher vertical velocities are necessary and larger forces occur [10, 11]. The variation among judges, however, becomes less relevant, the larger the data set. While our data set was comparably small, in studies that include a great amount of figure skating results, other parameters might be even more beneficial to study performance. The limitation of the present data set is the small amount of data that was due to the implementation of the new ISU Judging System in the season 2004/2005. The major strength of our study is that it is the first to report performance changes over time in elite figure skating in the singles categories of ladies and men.

## 5. Conclusions

The present study was the first to analyse performance and age trends in elite figure skating in the singles categories of ladies and men. We showed that at the European and World Championships, the numbers of jumps with fewer rotations decreased in favour of more difficult jumps with more rotations. These results indicate a recent increase in performance in figure skating. As jumps with more rotations are associated with impacts of greater magnitude and intensity, these recent developments should be monitored concerning injury rates and chronic degenerative damage in athletes, especially in the women who reach their peak performance level earlier than before.

## References

[1] Soligard T, Steffen K, Palmer-Green D, Aubry M, Grant ME, Meeuwisse W, et al. Sports injuries and illnesses in the Sochi 2014 Olympic Winter Games. Br J Sports Med 2015; 49: 441–7. doi: 10.1136/bjsports-2014-094538 25631542

[2] Fortin JD & Roberts D. Competitive figure skating injuries. Pain Phys. 2003;6,313–318. (no doi available). 16880878

[3] Porter EB. Common injuries and medical problems in singles figure skaters. Curr. Sports Med. Rep 2013; 12: 318–320. doi: 10.1249/JSR.0b013e3182a4b94e 24030306

[4] Porter EB, Young CC, Niedfeldt MW, and Gottschlich LM. Sport-specific injuries and medical problems of figure skaters. WMJ. 2007; 106, 330–334. (no doi available). 17970015

[5] Ridge S, Bruening D, Charles S, Stahl C, Smith D, Reynolds R, et al. IceSense Proof of Concept: Calibrating an Instrumented Figure Skating Blade to Measure On-Ice Forces. Sensors (Basel). 2020, 20, 7082. doi: 10.3390/s20247082 33321886 PMC7763340

[6] Han JS, Geminiani ET, Micheli LJ. Epidemiology of Figure Skating Injuries: A Review of the Literature. Sports Health 2018; 10: 532–537. doi: 10.1177/1941738118774769 29738281 PMC6204632

[7] Jederström M, Agnafors S, Ekegren C, Fagher K, Gauffin H, Korhonen L, et al. Determinants of Sports Injury in Young Female Swedish Competitive Figure Skaters. Front Sports Act Living 2021; 3:686019. doi: 10.3389/fspor.2021.686019 34222861 PMC8253259

[8] Naylor TA & Naylor S. Distribution and risk factors for stress fractures in competitive figure skaters and association with acute fractures. Phys Sportsmed. 2021; 1–5. doi: 10.1080/00913847.2021.1981748 34529544

[9] Burt LA, Groves EM, Quipp K, and Boyd SK. Bone density, microarchitecture and strength in elite figure skaters is discipline dependent. J Sci Med Sport. 2021; S1440-2440(21)00234-6. doi: 10.1016/j.jsams.2021.09.001 34607766

[10] Lockwood K & Gervais P. Impact forces upon landing single, double, and triple revolution jumps in figure skaters. Clin. Biomech. 1997; 12: S11. doi: 10.1016/s0268-0033(97)88322-2 11415711

[11] King DL. Performing triple and quadruple figure skating jumps: implications for training. Can J Appl Physiol. 2005; 30(6), 743–53. doi: 10.1139/h05-153 16485524

[12] King DL, Smith S, Higginson B, Muncasy B, and Scheirman G. Characteristics of triple and quadruple toe-loops performed during the Salt Lake City 2002 Winter Olympics. Sports Biomech. 2004; 3: 109–23. doi: 10.1080/14763140408522833 15079991

[13] Jaworski CA & Ballantine-Talmadge S. On thin ice: preparing and caring for the ice skater during competition. Curr Sports Med Rep 2008; 7: 133–7. doi: 10.1097/01.CSMR.0000319710.25675.1e 18477869

[14] Berthelot G, Tafflet M, El Helou N, Len S, Escolano S, Guillaume M, et al. Athlete atypicity on the edge of human achievement: performances stagnate after the last peak, in 1988. PLoS One 2010; 5: e8800. doi: 10.1371/journal.pone.0008800 20098706 PMC2808355

[15] Lippi G, Banfi G, Favaloro EJ, Rittweger J, and Maffulli N. Updates on Improvement of Human Athletic Performance: Focus on World Records in Athletics. Br Med Bull 2008; 87, 7–15. doi: 10.1093/bmb/ldn029 18723588

[16] Marck A, Antero J, Berthelot G, Saulière G, Jancovici JM, Masson-Delmotte V, et al. Are We Reaching the Limits of Homo sapiens? Front Physiol 2017; 8: 812. doi: 10.3389/fphys.2017.00812 29123486 PMC5662890

[17] Nevill AM & Whyte G. Are There Limits to Running World Records? Med Sci Sports Exerc 2005; 37: 1785–8. doi: 10.1249/01.mss.0000181676.62054.79 16260981

[18] Weiss M, Newman A, Whitmore C and Weiss S. One hundred and fifty years of sprint and distance running–Past trends and future prospects. Eur J Sport Sci 2016;16(4), 393–401. doi: 10.1080/17461391.2015.1042526 26088705 PMC4867877

[19] Ganse B & Degens H. Declining track and field performance trends in recent years in the Austrian best results 1897–2019. J Musculoskelet Neuronal Interact 2021; 21(2), 196–205. (no doi available). 34059565 PMC8185268

[20] Kruse TN, Carter RE, Rosedahl JK and Joyner MJ. Speed Trends in Male Distance Running. PLoS One 2014; 9(11), e112978. doi: 10.1371/journal.pone.0112978 25409192 PMC4237511

[21] Perneger TV. Speed trends of major cycling races: does slower mean cleaner? Int J Sports Med. 2010; 31: 261–264. doi: 10.1055/s-0030-1247593 20148370

[22] Gomez AT & Rao A. Adventure and Extreme Sports. Med Clin North Am 2016; 100: 371–91. doi: 10.1016/j.mcna.2015.09.009 26900120

[23] Lutter C, Tischer T, Schöffl VR. Olympic competition climbing: the beginning of a new era-a narrative review. Br J Sports Med 2021; 55: 857–864. doi: 10.1136/bjsports-2020-102035 33036996

[24] Steffen K, Soligard T, Mountjoy M, Dallo I, Gessara AM, Giuria H, et al. How do the new Olympic sports compare with the traditional Olympic sports? Injury and illness at the 2018 Youth Olympic Summer Games in Buenos Aires, Argentina. Br J Sports Med 2020; 54(3), 168–175. doi: 10.1136/bjsports-2019-101040 31796464

[25] Baker AB & Tang YQ. Aging performance for master records in athletics, swimming, rowing, cycling, triathlon, and weightlifting. Exp Aging Res 2010; 36: 453–77. doi: 10.1080/0361073X.2010.507433 20845122

[26] Donato AJ, Tench K, Glueck DH, Seals DR, Eskurza I, and Tanaka H. Declines in physiological functional capacity with age: a longitudinal study in peak swimming performance. J Appl Physiol. 1985; 2003(94), 764–9. doi: 10.1152/japplphysiol.00438.2002 12391125 PMC5063028

[27] Hoog Antink C, Braczynski AK, Kleerekoper A, Degens H, and Ganse B. Longitudinal master track and field performance decline rates are lower and performance is better compared to athletes competing only once. J Gerontol A Biol Sci Med Sci. 2020; 76(8), 1376–1381. doi: 10.1093/gerona/glab049 33606016

[28] Ganse B, Ganse U, Dahl J, and Degens H. Linear Decrease in Athletic Performance during The Human Life Span. Front Physiol. 2018; 9, 1100. doi: 10.3389/fphys.2018.01100 30246782 PMC6110907

[29] Website of the International Skating Union (ISU). Available from: https://www.isu.org/figure-skating/entries-results/fsk-results.

[30] Hirosawa S, Watanabe M, Aoki Y. Determinant analysis and developing evaluation indicators of grade of execution score of double axel jump in figure skating. J Sports Sci. 2021; 16: 1–12. doi: 10.1080/02640414.2021.1997407 34781855

[31] Schade F, Arampatzis A, Brüggemann GP, and Komi P. Comparison of the men’s and the women’s pole vault at the 2000 Sydney Olympic Games. J Sports Sci. 2004; 22(9), 835–42. doi: 10.1080/02640410410001675315 15513277

[32] Bruening DA & Richards JG. The effects of articulated figure skates on jump landing forces. J Appl Biomech 2006; 22: 285–95. doi: 10.1123/jab.22.4.285 17293625

[33] Pelham TW, Holt LE, Moss MA. Exposure to carbon monoxide and nitrogen dioxide in enclosed ice arenas. Occup Environ Med 2002; 59: 224–33. doi: 10.1136/oem.59.4.224 11934949 PMC1740267

[34] Pennanen AS, Salonen RO, Aim S, Jantunen M.J., and Pasanen P. Characterization of Air Quality Problems in Five Finnish Indoor Ice Arenas. J Air Waste Manag Assoc 1997; 47: 1079–1086. doi: 10.1080/10473289.1997.10464405 28445121

[35] Chapman RF, Stickford JL, Levine BD. Altitude training considerations for the winter sport athlete. Exp Physiol 2010; 95: 411–21. doi: 10.1113/expphysiol.2009.050377 19837773

[36] Meyer NL, Manore MM, Helle C. Nutrition for winter sports. J Sports Sci 2011; 29 Suppl 1: S127–36. doi: 10.1080/02640414.2011.574721 22150424

[37] Looney MA. Objective measurement of figure skating performance. J Outcome Meas 1997; 1: 143–63. (no doi available). 9661718

